# Positive cardiovascular health: longitudinal investigation of sustained health behavior in a cross-lagged model

**DOI:** 10.3389/fpubh.2024.1400849

**Published:** 2024-08-22

**Authors:** Zsofia Ocsovszky, Blanka Ehrenberger, Blanka Berenyi, Alexandra Assabiny, Jozsef Otohal, Tamas Martos, Orsolya Papp-Zipernovszky, Fanni Hegedus, Bela Merkely, Marta Csabai, Zsolt Bagyura

**Affiliations:** ^1^Heart and Vascular Center, Semmelweis University, Budapest, Hungary; ^2^Doctoral School of Cardiovascular Medicine and Research Program, Semmelweis University, Budapest, Hungary; ^3^Faculty of Humanities and Social Sciences, Institute of Psychology, Karoli Gaspar University of the Reformed Church, Budapest, Hungary; ^4^Faculty of Humanities and Social Sciences, Psychology Institute, University of Szeged, Szeged, Hungary

**Keywords:** positive cardiovascular health, health awareness, health behavior change, positive psychology, cardiovascular health prevention

## Abstract

**Objective:**

Our study focuses on the role of psychological states in the development of cardiovascular disease (CVD) and explores the potential of positive psychological factors in reducing CVD risk. While existing research has predominantly examined negative mental states and risk behavior, this longitudinal study takes a novel approach by investigating positive psychological wellbeing and its impact on sustained health behavior.

**Method:**

The research involved participants (*n* = 502) with medium to high cardiovascular risk who underwent a comprehensive risk assessment in 2012, followed by written risk communication. Health behavior and psychological variables were measured in 2012 and 2019. A cross-lagged panel was employed to repeat measures of a cardiovascular health index with latent factors.

**Results:**

Results indicated an excellent fit for the model (RMSEA = 0.0644, CFI = 0.936, TLI = 0.921, SRMR = 0.050), with significant associations between the observed variables (*p* < 0.05) and created latent factors. Furthermore, the model implied significant bivariate correlations (*p* < 0.05) between latent constructs of sustained health behavior and positive psychological states in 2012 and 2019. A significant regression relationship between Health Awareness Index 2012 and 2019, between Psychological wellbeing in 2012 and 2019 (B = 1.103 *p* = 0.038), latent factors could be identified (B = 1.103 *p* = 0.038) using cross-lagged panel model. Results highlighted the importance of cardiovascular health awareness, subjective risk perception, and self-directed efforts in facilitating health behavior change.

**Conclusion:**

Relationships between psychological wellbeing and health awareness emphasize that positive experiences and reinforcement are crucial in sustaining optimal health behavior. Our findings offer a new perspective on cardiovascular risk screening and preventive interventions. Extending cardiovascular risk screening with psychological measures may broaden prevention opportunities by including psychological elements reinforcing positive psychological states. The findings suggest that an effective prevention program must consider stabilizing and maintaining positive psychological states to achieve lasting improvements in cardiovascular health.

## 1 Introduction

Despite worldwide initiatives, including research, technological advancements, and economic contributions, the prevalence of cardiovascular diseases (CVD) continues to escalate. The European Society of Cardiology (ESC) 2019 Fact Sheet reveals that, in Europe, 6 million new cases were registered, and globally, 11 million, impacting a total of 49 million individuals and leading to 3.9 million fatalities. The medical treatment of these patients constitutes a considerable economic strain, and a substantial number of individuals succumb to this preventable chronic illness (1).

Despite significant progress in treatment options and prevention strategies, CVD remains a major global health challenge (2, 3). A potential explanation of this health burden is the complex nature of CVD. There are numerous risk factors for CVD, such as smoking, unhealthy diet, physical inactivity, and genetic predisposition.

The 2021 ESC CVD prevention guideline (4) lists classic influencing factors such as cholesterol levels, smoking, blood pressure, diabetes, and obesity, as well as psychosocial factors among the risk influencers. There is extensive literature on the direct impact of mental states and their indirect effects on health behavior. Affective and anxiety disorders [depression (5, 6), anxiety (6), and PTSD (Post Traumatic Stress Disorder)] (7) have been proven by numerous research groups to have a direct influence on CVD and to worsen outcomes. Rumination, negative emotions, and hostility exert their detrimental effects through health representations, self-efficacy, and other behavioral factors leading to maladaptive health behaviors (8–10). Havranek et al. (11), in their statement published in the journal Circulation, specifically emphasize the influential power of social factors and provide an integrative framework by expanding the WHO definition of Social Determinants of Health (SDOH) as “the circumstances in which people are born, grow, live, work, and age, and the systems put in place to deal with illness” (12) with psychological, behavioral, and biological mechanisms that trigger and perpetuate cardiovascular diseases.

Therapeutic and technological advances have prevented many deaths over the past 30 years. However, these patients continue to live with chronic conditions that place a significant burden on the healthcare system, the economy and their quality of life.

Long-term CVD cases can only be reduced through well-planned and optimized preventive measures. This requires a more precise understanding of the pathways of influencing factors and the incorporation of new research paradigms.

As explained above, literature reviews on the psychosocial determinants of CVD mainly focus on negative emotional states (primarily depression and anxiety), negative personality traits such as anger, hostility, and pessimism, and chronic and acute stressors, including work stress and social isolation. However, in recent years the importance of a positive psychological approach to CVD has been increasingly recognized.

In the late 80s, Martin Seligman and Mihaly Csikszentmihalyi introduced positive psychology as a new paradigm in the field, emphasizing the scientific study of positive human functioning (13), and later on, in 2008, Seligman extended the concept by proposing a multidimensional approach to positive health underscoring subjective, biological, and functional dimensions as predictors of overall wellbeing (14). Further research has been conducted to support the theory that positive psychological characteristics, including but not limited to happiness, optimism, gratitude, sense of purpose, life satisfaction, and mindfulness, are linked to a reduced risk of cardiovascular disease and mortality (15–18). In one of the largest and most comprehensive systematic reviews on this topic to date, researchers found that positive psychological wellbeing appears to reduce the risk of heart attacks, strokes, and other cardiovascular events (19). The statement of the American Heart Association (20) synthesized the knowledge on the effect of psychological factors and emotional states on CVD, highlighting that positive psychological traits, such as optimism, positive outlook, and having a purpose in life, significantly reduced the risk of a heart attack by respectively 38, 32, and 38%.

Research (21) indicates that practices like mindfulness, which involves staying present with openness and nonjudgment, learning calmness, and stress management skills, can effectively reduce CVD. Additionally, related mind-body techniques have been utilized to enhance psychological wellbeing. For a positive state of mind and subjective psychological wellbeing, one might experience the integrity of mind and body founded by health. A sense of satisfaction with health behavior facilitates engagement in such practices (22).

These findings support the idea that positive psychological states are crucial in facilitating long-term changes in health behavior (23). Positive psychology can be an effective approach to health promotion and prevention and treatment of CVD. Positive emotions can encourage the development and maintenance of healthy behaviors, contributing to the success of long-term, sustainable health promotion strategies (22, 24, 25).

As described above, negative mental states can influence unhealthy behaviors through cognitive processes. In contrast, positive psychological states and a higher level of overall subjective wellbeing contribute to health-promoting behaviors (26). Following this paradigm, our hypothesis that psychological wellbeing is also associated with the improvement and long-term sustainability of CVD preventive health behaviors, thus serving as a starting point for preventive interventions. Based on this approach, in our follow-up study, we aim to examine the contribution of psychological wellbeing to long-term health awareness in a Structural Equation Model (SEM).

## 2 Materials and methods

**Figure 1:**
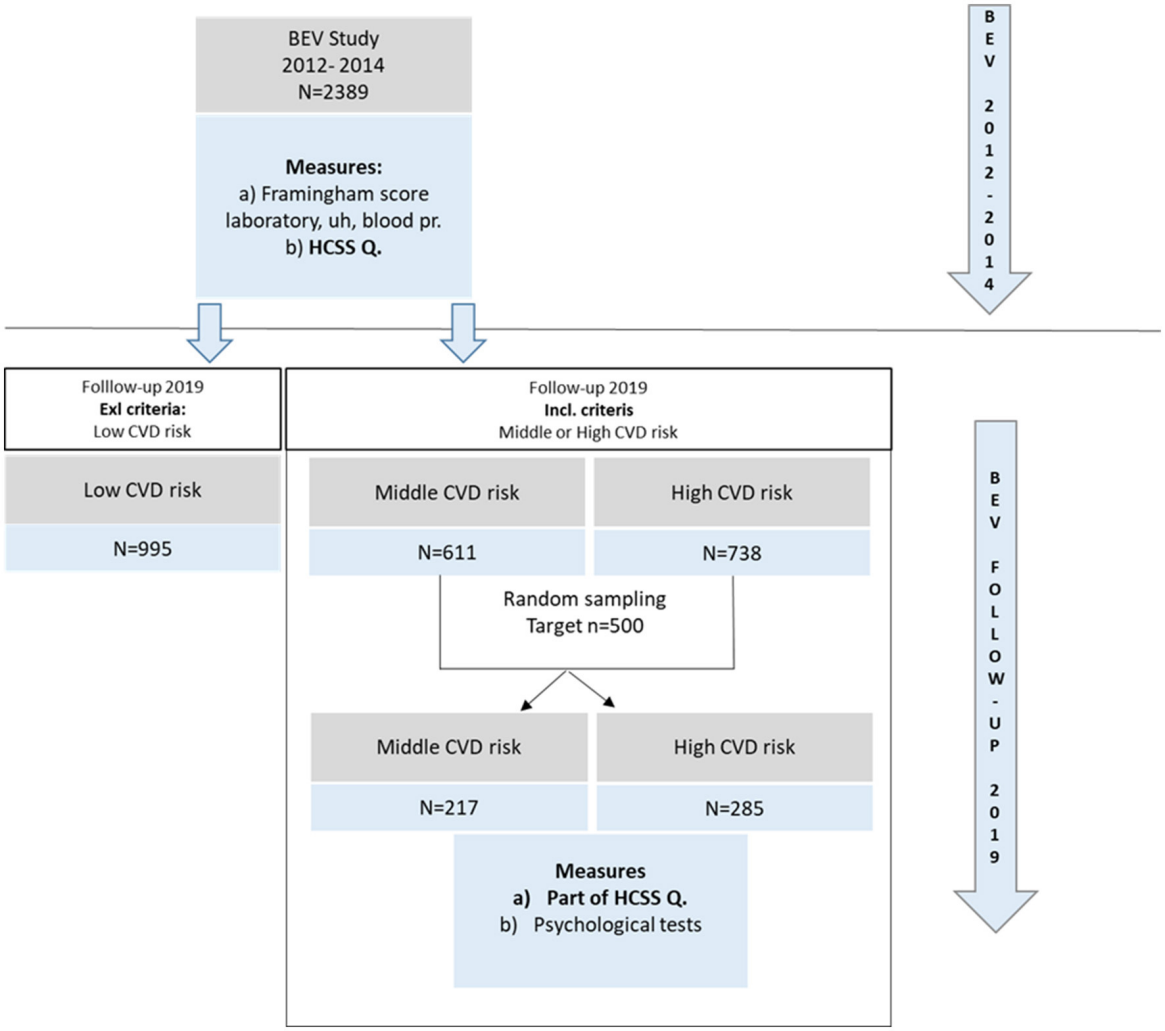
Selection process.

The current study was preceded by a comprehensive voluntary-based cardiovascular screening program for the adult population (26), beginning in 2012, called Budakalász Epidemiological Study (BES), consisting of (1) a health questionnaire (developed by the Hungarian Center of Social Sciences (HCSS) for the European Health Interview Survey (EHIS), (2) non-invasive tests (anthropometric measurements, echocardiography, carotid artery ultrasound, blood pressure measurement, ankle-brachial index measurement), as well as (3) venous blood sampling and laboratory examinations. By January 2014, 2,389 individuals had undergone physical examinations and cardiovascular risk assessments using the Framingham risk scale (27). Following the initial Budakalász baseline study between 2012 and 2014, repeated administration of selected items of the baseline questionnaire mentioned above (EHIS) and further psychological data collection was conducted in 2019. The data has been gathered by a professional company specializing in psychological and sociological data collection. Their interviewers have attended internal training on this study, its requirements and methodology. All tests and measures administered are validated, standardized questionnaires in national and Hungarian samples. The research aim was to follow up with at least 500 individuals through random sampling. Thus, the current sample (*n* = 502) was selected from the 2012 cohort (*n* = 2,389). The inclusion criteria were set for medium and high cardiovascular risk based on the Framingham scoring method. Of the 1,394 individuals initially characterized as medium or high cardiovascular risk, 502 were contacted. The selection process is illustrated in Figure 1. Preliminary awareness was raised through the local Health Club and local newspaper, along with an educational presentation about the study and cardiovascular diseases, to increase the response rate. During data cleaning, the data collected in 2012–2014 and 2019 were matched based on personal data. Power analysis was completed to confirm the sample size (see below under 3.2).

### 2.1 Measures

**A health awareness index (HAI)** was developed in our previous research (28) and used as a measure of health behavior. The items for the index were selected from the questionnaire of the Hungarian Center of Social Sciences (HCSS), a measurement tool consisting of 128 items. The Hungarian Central Statistical Office used this questionnaire during the European Health Interview Survey (EHIS) (29). We have administered it during our two data collection processes (2012–2014- baseline; 2019- follow-up). Items used for HAI: (1) How would you generally describe your health? (very bad, bad, satisfying, good, and very good) (2) How much do you think you can do for your health? (nothing, not too much, much, and very much) (3) How many days have you engaged in intense physical activity in the past seven days? (4) How often do you consume vegetables and fruits? (less frequently than once a week, at least once a week, at least four times a week, daily, multiple times a day).

The Short Form (36) **Health survey** is a 36-item, patient-reported survey of patient health.

As part of the EHIS questionnaire, it was administered as a baseline (2012–2014 and as a follow-up in 2019). In 2019, four of the eight mental health/psychological wellbeing scale items were selected based on a preliminary scale analysis of the BES dataset to measure psychological wellbeing. Items were prompted as follows: “In the past 2-4 weeks, how often did you feel (1) calm and peaceful, (2) energetic, (3) happy, (4) tired?” Items were rated on a 5-point scale ranging from 1 = never to 5 = always, the “tired” with reversed scoring. In our sample, the internal reliability of the scale was adequate in terms of the expected correlation value between the items (Cronbach's alpha of 2012/2019: 0.624/0.782).

### 2.2 Statistical methods

We have used SEM, which encompasses a set of various methods. SEM includes building a model, an informative representation of an observable or theoretical phenomenon. In this model, different aspects of a phenomenon are theoretically constructed to be interconnected with a structure (30–36). SEM is comparable yet more potent than regression analyses; this method investigates linear causal connections between variables while also considering measurement error. SEM offers a fresh viewpoint for data analysis and the potential to enhance medical and health science research (37).

We also aimed to use power analysis to ensure sufficient sample size to create a stable model. Statistical power is a concept arising in the context of classical null hypothesis significance testing, where a null hypothesis (H0) is evaluated against an alternative hypothesis (H1). In any hypothesis test, two types of decision errors may occur: the alpha error of incorrectly rejecting a true null hypothesis (and thus wrongly accepting a false alternative hypothesis) and the beta error of incorrectly retaining a false null hypothesis (and thus incorrectly rejecting a true alternative hypothesis). Statistical power complements the beta error and gives the probability of rejecting a null hypothesis if this hypothesis is factually wrong (and thus to accept a true alternative hypothesis) (38).

The statistical analyses were performed using JAMOVI 2.4.11 (39) statistical software and semPower (38). For an integrated analysis of health behavior, we created a Health Awareness Index and applied discrete-time structural equation modeling, with a particular emphasis on cross-lagged relationships. Based on our previous results (40), we aimed to investigate the changes in factors constituting health awareness and their relationship with psychological characteristics, as well as a more thorough analysis of the cardiovascular risk assessment and the health awareness factor. This method allows for the inclusion of various causes and outcomes, lowers the risk of Type I error compared to one- or two-variable testing, allows for the possibility of refining relationships between variables, reduces the impact of measurement error, and allows for advanced handling of missing data (41), thereby enabling a more integrated and extended approach to long-term health behavior and mental wellbeing.

The internal consistency of psychological tests for this population was checked with a reliability test, and the value was reported in Cronbach's Alpha.

We based our study on the Health Awareness Index (HAI) we developed in our previous research to investigate changes in health consciousness. Considering all this, we defined and examined a latent health consciousness variable and a psychological wellbeing variable characterized by manifest variables at two points, 2012 and 2019.

Description of the latent variables

1. The latent psychological wellbeing variable was determined by selecting four items from the SF-36 wellbeing questionnaire. In the past 4 weeks, how often did you feel (1) calm and peaceful (2) energetic (3) happy and (4) tired?
2. Components of latent HAI variables (2012, 2019): (1) How would you generally describe your health? (subj health) (2) How much do you think you can do for your health? (health action) (3) In the past 7 days, how many days did you engage in intense physical activity? (activity) (4) How often do you consume vegetables and fruits? (veg&fruits)

A *p*-value of <0.05 was considered statistically significant in the conducted tests.

## 3 Results

### 3.1 Power analysis

An a priori power analysis was conducted to determine the minimum sample size required to test the model. Results indicated the size of the sample needed to achieve 97% power for detecting the effect size RMSEA = 0.044, at a significance criterion of α = 0.05, df = 94 with 16 manifest variables. We got 333 as the required sample size. The sample size used for the SEM model (*N* = 502) is adequate for testing the research model. Based on the elimination of the second-order error, there are no further significant correlations beyond the found significant correlations.

### 3.2 Descriptive

From the initial Budakalász database, 1,394 people were selected in the sample, which includes 892 unfollowed cases and 502 followed cases. We found no significant differences in socio-demographics, health behaviors and indicators when comparing them (see Supplementary Table). Marital status was an exception according to X2 test (X2 = 12.6;4; (N = 1392), = 12.6 *p* < 0.05), a significant difference with a low degree of effect size (Cramer's V = 0.0953) can be evidenced between the two groups, meaning that in the followed subsample there were relatively less married (between those who live together or married and those who live separately or divorced than in the not followed subsample (Table of Comparison of followed and not followed subsamples' characteristics of Budakalász baseline study can be seen as Supplementary material). For the SEM model, the sample consisted of 502 followed people with medium and high cardiovascular risk. The risk was assessed using the Framingham assessment tool. Two hundred and seventeen people (43%) have a medium, and 285 people (57%) have a high cardiovascular risk. Based on the gender distribution, 225 men (45%) and 277 (55%) women between the ages of 45 and 98 were included in the study. The average age was 71 ± 8.57 years. The majority of the population was single (*n* = 277; 55.17%) and had primary (*n* = 178; 35.45%) and secondary (*n* = 174; 34.66%) education.

As the first step of the Structural Equation Model, we defined the individual latent variables and then determined the relationships between these variables (please see the description in the Section 2.2: Description of the latent variables).

Characteristics of the latent health awareness factor and SF-36 of 2012 and 2019 are contained in Supplementary Table 1.

### 3.3 Model settings

**Figure 2:**
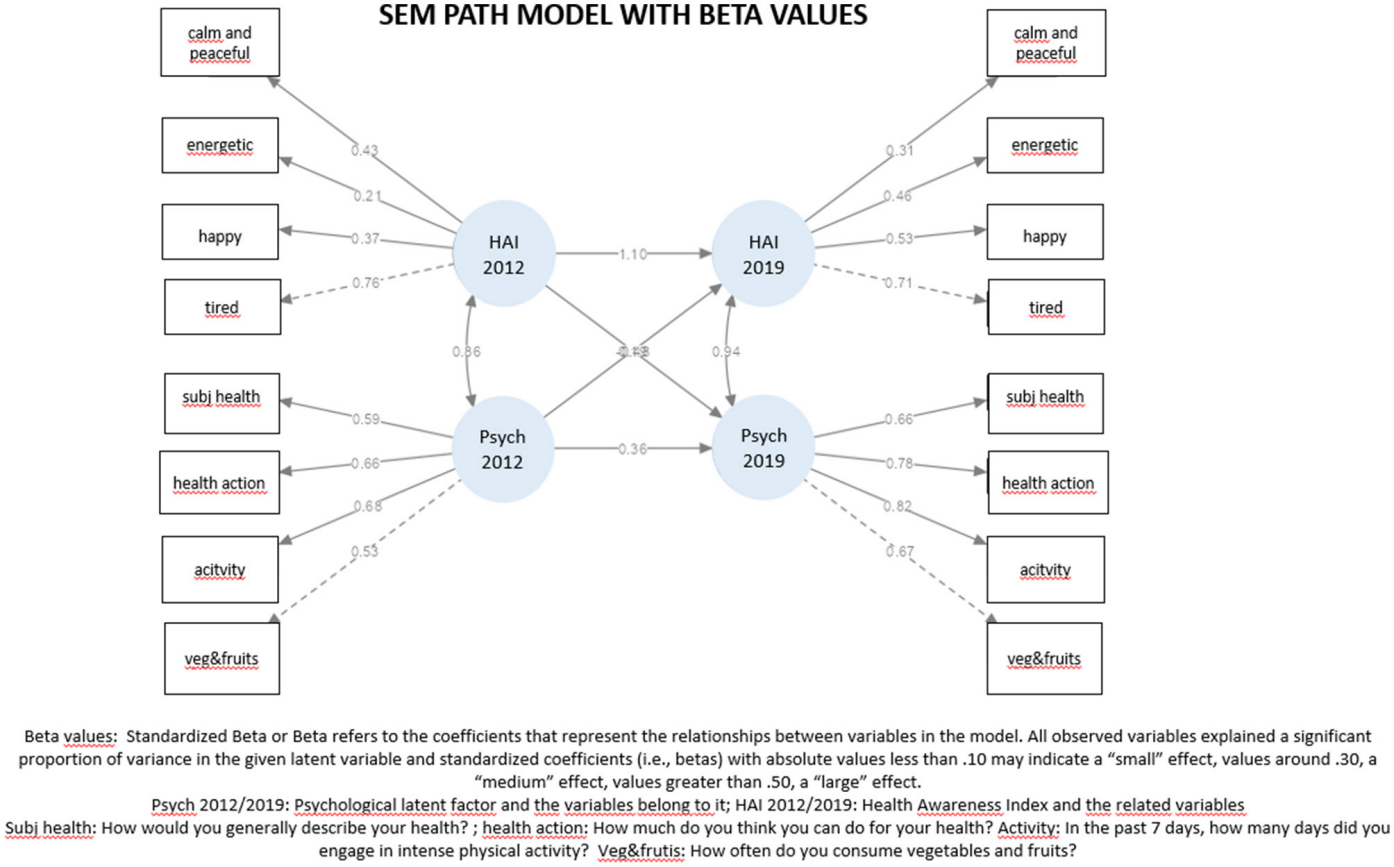
SEM model.

Because the variables did not adhere to a normal distribution, the testing was conducted using robust methods. Robust method is a statistic that retain their properties even when the underlying distributional assumptions are incorrect. We posited a linear regression connecting Health Awareness in 2019, Health Awareness in 2012, and Psychological wellbeing in 2012. Similarly, we conjectured a relationship involving Psychological wellbeing in 2019, Health Awareness in 2012, and Psychological wellbeing in 2012. Furthermore, we determined a covariance relationship between the Health Awareness in 2012 and Psychological wellbeing in 2012 latent variables and between the Health Awareness in 2019 and Psychological wellbeing in 2019 latent variables. The model is depicted in Figure 2.

### 3.4 Model evaluation

#### 3.4.1 Goodness-of-fit index

The Fit of the model was evaluated with the following indices and indicators (the limit values of the good indicators are given in parentheses). Comparative Fit Index (CFI >0.90), Tucker-Lewis index (TLI >0.90), Root Mean Square Error of Approximation (RMSEA>0.10), Standardized Root Mean Squared Residual (SRMR >0.08), Goodness of fit (GFI >0.90) (42). The Chi-square test (χ^2^ (984) = 286 *p* < 0.05) indicates a significant difference, but this value is disregarded for the estimation of model fit, as it is difficult to achieve a good fit in a large sample using χ^2^ (43). Based on the fit indicators, the model has an adequate fit: CFI = 0.936, TLI = 0.921, RMSEA = 0.064, SRMR = 0.050, GFI = 0.985.

### 3.5 The main results of the measurement model and structural model

#### 3.5.1 Measurement model

Supplementary Table 3 presents the main results of the measurement model. Estimates, standardized estimates, Beta Coefficients, and *p*-values for the measurement model are reported with regard the observed variables. According to the significance levels, all the observed variables have a significant role in the given latent variable. Beta Coefficients point out how strong the effect of the given variable is. Standardized Beta or Beta refers to the coefficients representing the relationships between model variables. These coefficients are crucial in SEM as they indicate the strength and direction of the effects of observed variables on latent variables in the model. All observed variables explained a significant proportion of variance in the given latent variable and standardized coefficients (i.e., betas) with absolute values less than .10 may indicate a “small” effect, values around .30, a “medium” effect, values >0.50, a “large” effect.

Bivariate correlations (standardized covariance estimates) of the latent variables are reported in Supplementary Table 4. We found highly significant (*p* < 0.001) positive associations between all latent constructs, ranging from 0.47 to 0.84, indicating their multiple interconnectedness.

#### 3.5.2 Path model

**Figure 3:**
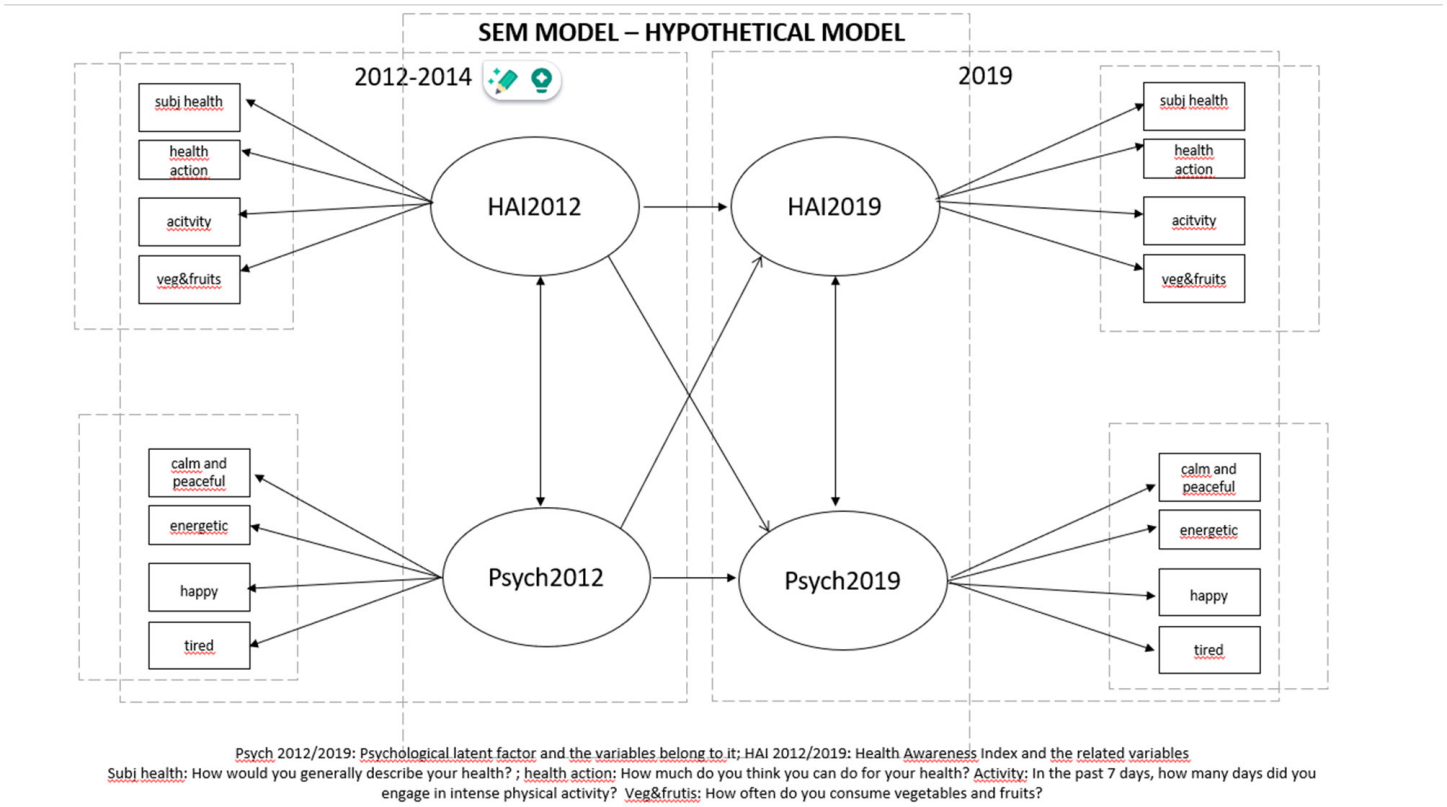
Path model—path coefficient estimates.

We tested the predictive associations between the psychological and health awareness constructs from 2012 to 2019 in a cross-lagged panel model. Path coefficient estimates, standardized estimates, and *p*-values in the final path model are reported in Supplementary Table 5. Based on the significance values, it can be concluded that the latent factor Health Awareness Index in 2012 directly predicts the Health Awareness Index in 2019 (beta = 1.10, *p* = 0.038), and the Psychological Measure 2012 directly predicts the Psychological Measure 2019 (beta = 0.36, *p* = 0.037) (Supplementary Table 4). The cross-lagged predictions did not reach significance. The model is depicted in Figure 3.

## 4 Discussion

The results partially support our hypothesis on the relationship between wellbeing and sustained health behavior. Although we did not find a significant regression relationship between all the hypothesized latent factors, the Health Awareness Index in 2012 does influence the Health Awareness Index in 2019, and psychological wellbeing in 2012 does influence psychological wellbeing in 2019. In addition, the goodness of our theoretical model was adequate, and there are medium to strong positive bivariate associations between the latent factors. It suggests that a well-founded cardiovascular health awareness and an increase in the level of subjective risk are the starting points in health behavior change. This awareness and self-direction, along with increased internal control, can enhance psychological wellbeing, which jointly supports the maintenance of healthy attitudes and behavior. Several researchers have explored Seligman's positive health concept (14) in depth. We aimed to contribute to this initiative by studying health behavior from a positive psychological perspective. While most studies in this field have used cross-sectional methods, only a few have employed SEM methodology, and even fewer have conducted longitudinal studies with repeated measurements. We aimed to utilize every possible approach to better understand the problem and provide further evidence to support this concept. In our SEM model, we could not identify the cross-lagged predictive properties of previous levels of wellbeing and health behavior on later levels of the other characteristics, as did Boehm's review (44), which comprehensively presents numerous studies on this topic. Some of them prove that consumption of vegetables and fruits and physical activity are associated with wellbeing, and lower levels of positive states with risk behaviors. These results demonstrate that in the case of a preventive intervention, it is essential to consider the psychological status of the individual/patient. Without reinforcing, improving, and maintaining positive psychological factors, we cannot expect the development of long-term sustained adaptive health behaviors, which are of primary importance from a CVD perspective. We must highlight the main difference between these studies and our investigation. While we employed SEM in a cross-lagged model and longitudinal setting, the studies in Boehm's review proceed with the cross-sectional method.

As discussed before, most evidence is based on cross-sectional methodology, and the underlying research shows a considerable variety of methods, but two of them (45, 46) regarding physical activity that use longitudinal setting support our theory and results. Lee's (47) publication investigates physical activity, self-rated health, and psychological resilience in a repeated measure SEM model with two follow-up points. Her findings show that physical activity and resilience were associated positively with self-rated health over time, but no significance was found between physical activity and resilience.

Our SEM model has an adequate fit supporting the concept of positive health by presenting wellbeing and health awareness as being separate and still interrelated constructs at the same time. This result follows the study of Stenlund et al. (41). In their research, they applied the same methodology (working with latent variables, using longitudinal SEM) as our team, emphasizing the mutual relationship between wellbeing and health behavior, confirming our expectations about the predictive power of health behavior on later wellbeing, despite us not finding a significant relationship in our model. An additional difference is that in our research, in contrast to theirs, health awareness (HAI) is a more complex construct because, beyond health behavior, it encompasses attitudes toward health, which measure readiness to act, thereby enabling a more dynamic and proactive assessment.

The present study aimed to investigate the impact of positive psychology on sustained health behavior in the context of cardiovascular disease (CVD) prevention. Using a structural equation modeling (SEM) approach, we comprehensively analyzed repeated measures within an extended framework to understand these variables' interplay. Our research findings align with the recommendations outlined in the American Heart Association's (AHA) Statement on Psychological Health, Wellbeing, and the Mind-Heart-Body Connection (2021). Therefore, we intend to expand our study to investigate the constructs of positive psychology and their relationship with CVD more extensively. Our research group believes this will contribute to the global and European CVD prevention objectives. We hope this research will offer fresh insights into CVD prevention and inspire future research in this field.

**Applicability of our results:**

Our results show that we have created a well-functioning model (see model indicators) in which the observed variables build up the subjective positive psychological states and sustained health behavior latent variables; however the assumed bi-directional effects still need to be fulfilled. In cardiovascular risk prevention, the golden standard interventions focus on changing health behavior (reducing the risk behavior and increasing the preventive behaviors) in the long term. Although our results indicates that it can only be feasible while maintaining sufficient psychological states simultaneously. Thus, our result offers a new perspective on cardiovascular risk screening with psychological measures might widen the preventional possibilities by including psychological elements reinforcing positive psychological states.

### 4.1 Limitations

Researchers aiming to comprehend the links between positive psychology and beneficial health outcomes should investigate various influencing factors (48). In our research, a limited number of factors were available for examination in a positive framework. Consequently, despite emphasizing that our model shows a good fit, we could not demonstrate a direct effect between the latent variables and the currently involved variables. In addition, most of the variables constituting health awareness are qualitative variables, and with scale variables, much more varied results can generally be obtained. Another limitation is that we have preliminarily focused on people with medium to high CVD risk. We did not include individuals with low risk in the study. All of this determines the linear regression relationships.

In future research, it will be necessary to include a variety of positive psychological variables and individuals with low CVD risk, as well as more quantitative variables regarding health consciousness.

## 5 Conclusion

Understanding the development of cardiovascular diseases (CVD) requires a holistic approach to designing complex preventive interventions. Psychological factors can impact the heart and cause somatic changes. Previous studies have identified several factors that increase CVD risks, such as depression, anxiety, and other mental issues. However, adopting a positive psychological approach can offer new perspectives on how to prevent CVD. This paradigm shift may require the application of more complex methodologies to reflect a holistic perspective. Our research aimed to introduce new methods for understanding the pathways of sustained health behavior change. While it did not confirm that previous psychological wellbeing and health awareness may contribute to long-term changes in wellbeing and health behavior, it did provide support for the assumption of a bidirectional association between these characteristics at the cross-sectional level. According to previous findings, the positive experience and reinforcement of efforts to improve health are crucial for maintaining optimal health behavior. Without stabilizing and maintaining such psychological states, it is impossible to design a valid prevention program to prevent CVD. Further research should be conducted to gain a more comprehensive understanding of the subject matter.

## Data Availability

The raw data supporting the conclusions of this article will be made available by the authors, without undue reservation.
