# Supplementary material for: Positive cardiovascular health: longitudinal investigation of sustained health behavior in a cross-lagged model
Source: Front Public Health. 2024 Aug 22;12:1400849. doi: 10.3389/fpubh.2024.1400849 (PMC11391933; doi:10.3389/fpubh.2024.1400849)
Supplement: Supplementary file 1 [file Table_1.pdf]

Supplementary Materials

Table 1. Descriptive statistics of the variables of the Health Awareness Index 2012 and 2019 latent variables

	2012		2019	
	**Subjective Health**			
	N	%	N	%
very bad	6	1,2	16	3,2
bad	43	8,6	70	13,9
satisfying	218	43,7	271	54
good	206	41,3	137	27,3
very good	26	5,2	8	1,6
**Total**	499	100	502	100
	**Health Action**			
	N	%	N	%
nothing	6	1,2	8	1,6
few	72	14,6	73	14,7
a lot	367	74,4	350	70,4
very much	48	9,7	66	13,3
**Total**	493	100	497	100
	**Physical Activity**			
**Days**	N	%	N	%
0	400	79,7	115	23
1	27	5,4	29	5,8
2	17	3,4	58	11,6
3	18	3,6	53	10,6
4	5	1	31	6,2
5	4	0,8	46	9,2
6	5	1	8	1,6
7	26	5,2	161	32,1
**Total**	502	100	501	100

	Fruit & Vegetable Consumption			
	N	%	N	%
less than once a week	2	0,4	2	0,4
at least once a week	25	5	33	6,6
at least four times a week	75	15	95	19
once a day	304	60,7	192	38,4
2/3 per day	95	19	178	35,6
**Total**	501	100	500	100

Table 2. Descriptive statistics of the variables of the psychological well-being latent variables of 2012 and 2019

	2012		2019	
	**Calm and peaceful**			
	N	%	N	%
1	4	0,7	14	2,8
2	114	22,8	74	15
3	174	34,7	107	21,6
4	195	39	196	39,6
5	14	2,8	104	21
**Total**	501	100	495	100
	**Energetic**			
	N	%	N	%
1	2	0,4	25	5,1
2	42	8,4	99	20
3	96	19,2	136	27,5
4	333	66,6	156	31,5
5	27	5,4	79	16,9
**Total**	500	100	495	100
	**Happy**			
	N	%	N	%
1	1	0,2	8	1,7

2	52	10,4	71	14,6
3	184	36,8	120	24,7
4	249	50	177	36,4
5	13	2,6	110	22,6
**Total**	499	100	486	100
		**Tired**		
	N	%	N	%
1	9	1,8	44	8,9
2	137	27,3	156	31,5
3	222	44,3	160	32,3
4	127	25,3	110	22,2
5	6	1,2	25	5,1
**Total**	501	100	495	100

Note: Never (1), Sometimes (2), Often (3), Constantly (4)

Table 3. Measurement Model

Latent	Observed	t	z	β	p
Psych2019	X1	1.000		0.672	
	X2	1.219	21.38	0.819	< .05
	X3	1.157	20.30	0.777	< .05
	X4	0.988	19.88	0.664	< .05
Psych2012	X1	1.000		0.526	
	X2	1.294	13.67	0.680	< .05
	X3	1.255	13.39	0.660	< .05

	X4	1.123	13.12	0.590	< .05
HAI2019	X5	1.000		0.706	
	X6	0.753	14.13	0.531	< .05
	X7	0.649	13.93	0.458	< .05
	X8	0.434	10.05	0.306	< .05
HAI2012	X5	1.000		0.758	
	X6	0.491	11.10	0.373	< .05
	X7	0.279	6.97	0.212	< .05
	X8	0.564	10.66	0.428	< .05

Abbreviations: HAI: Health Awareness Index; Psych: Psychological measure

Note: Estimates (t), Standardized estimates (z), Beta Coefficient (β), Significance level (p); Standardized path coefficients with absolute values less than .10 may indicate a "small" effect, Values around .30, a "medium" effect, Values greater than .50, a "large" effect

Table 4. Standardized covariances between the latent variables

**Latent variables**		**Covariance**	**St. estimate**	**z**	**p**
HAI2019	HAI2012	0.37	0.69	12.66	< .001
Psych2012	HAI2012	0.34	0.86	13.68	< .001
Psych2019	HAI2012	0.25	0.50	12.75	< .001
Psych2019	Psych2012	0.18	0.52	12.39	< .001
Psych2012	HAI2019	0.17	0.47	9.57	< .001
Psych2012	HAI2019	0.40	0.84	18.77	< .001

Abbreviations: HAI: Health Awareness Index; Psych: Psychological Measure

Note: Standardized estimates (z) Significance level (p)

Table 5. Parameter estimates

Predictor	Dependent	t	z	β	p
HAI2012	HAI2019	1.207	2.076	1.103	0.038
Psych2012	HAI2019	-0.643	-0.996	-0.479	0.319
HAI2012	Psych2019	0.165	1.028	0.168	0.304
Psych2012	Psych2019	0.464	2.088	0.362	0.037

Abbreviations: HAI: Health Awareness Index; Psych: Psychological Measure

Note: Estimates (t) Standardized estimates (z), Beta Coefficient (β), Significance level (p)

Supplementary Table: Comparisons

Comparison of followed and not followed subsamples' characteristics in Budakalász baseline study						
		**Laboratory measuers**				
		**Not followed (N = 892)**		**Followed (N = 502)**		
		**M**	**SD**	**M**	**SD**	**U**
**Cholesterol**		**5.70**	**1.24**	**5.70**	**1.15**	**222363 (n.s.)**
**HDL**		**1.38**	**0.45**	**1.38**	**0.4**	**219992 (n.s.)**
**LDL**		**3.36**	**1.28**	**3.44**	**1.16**	**216957 (n.s.)**
**Glucose**		**6.16**	**1.61**	**6.30**	**2.11**	**223430 (n.s.)**
		**Sociodemographic, health behavior factors**				
		**X2**		**df**		**V**
**Education**		**11.70**		**9**		
**Marital status***		**12.60***		**4**		**0.0953**
**How would you generally describe**		**7.68**		**4**		

**your health? (subj. health)**			
**How much do you think you can do for your health? (health action)**	**0.25**	**3**	
**Irregular heartbeat**	**0.17**	**1**	
**Other heart disease**	**0.37**	**1**	
**Hypertonia?**	**1.69**	**1**	
**Diabetes?**	**0.07**	**1**	
**High cholesterol?**	**0.04**	**1**	
**Thrombosis?**	**0.84**	**1**	
**Smoking**	**0.39**	**2**	
**Alcohol consumption**	**0.33**	**1**	
**Income**	**15.1**	**10**	

Notes: *p<0,05, **p<0,01, ***p<0,001; n.s.: p>=0.05

Abbreviations: M = Mean; U = Mann–Whitney U-test; SD = standard deviation, X2 = chi-square test value, df = degree of freedom, V = Cramér's V

Only the medium and high CVD risk cases from the Budakalász 2012 sample
